# Why does COVID-19 continue to spread despite mass vaccination?

**DOI:** 10.3389/fpubh.2022.938108

**Published:** 2022-07-25

**Authors:** Shuo Zhang, Zhen Yang, Zhen-Lin Chen, Shi-Jun Yue, Sai Zhang, Yu-Ping Tang

**Affiliations:** ^1^Key Laboratory of Shaanxi Administration of Traditional Chinese Medicine for TCM Compatibility, Shaanxi University of Chinese Medicine, Xi'an, China; ^2^School of Clinical Medicine (Guang'anmen Hospital), Beijing University of Chinese Medicine, Beijing, China; ^3^School of Public Health, Shaanxi University of Chinese Medicine, Xi'an, China; ^4^International Programs Office, Shaanxi University of Chinese Medicine, Xi'an, China

**Keywords:** COVID-19, SARS-CoV-2, vaccine, vaccination, infection

## Introduction

COVID-19 is a severe respiratory disease that broke out in late 2019 (1), It is highly infectious and pathogenic which caused by SARS-CoV-2 infection (2). Since the outbreak, COVID-19 has spread almost rapidly around the world, the rapid increase in the number of patients has raised the alarm of the World Health Organization and has been classified as a public health emergency of international concern by the World Health Organization. As of 24 June 2022, COVID-19 has caused 546,550,738 confirmed infections and 6,347,660 deaths worldwide. Beyond the health impact, the COVID-19 pandemic has caused social, economic and political damage. At present, the overall situation of the epidemic is still serious, and mask wearing, social distancing, isolation and symptomatic support treatment are still the main priorities. With the rapid development of the COVID-19 epidemic, the emergence of mutant strains, and the lack of specific and effective treatments, there is growing expectation for a COVID-19 vaccine, and most people believe that vaccination essentially protects people from SARS-CoV-2 infection, preventing further spread of COVID-19, and that herd immunity following mass vaccination can further prevent outbreaks (3). However, COVID-19 vaccines have been mass administered in most countries at present, COVID-19 has not gone away. Outbreaks and epidemics continue from time to time, and the number of confirmed cases and deaths from it continues to rise. Why does COVID-19 persist despite mass vaccination, causing huge confusion among health care workers.

## Current situation of COVID-19 vaccines development and injection in the world

According to the data released by World Health Organization (WHO), 182 COVID-19 vaccines have entered the pre-clinical trial stage, of which 73 have entered the clinical research stage, and 21 have entered the clinical phase III or phase I II/III (4). Depending on the target and the technology used in preparation, vaccines can be divided into the following categories: inactivated vaccines, recombinant spike vaccines, viral vector vaccines, RNA vaccines, live attenuated vaccines, virus-like particles vaccines, and so on (5–7). Vaccinating as many people as possible against effective COVID-19 vaccines to achieve herd immunity as quickly as possible to prevent the pandemic. COVID-19 vaccines are now available in most countries around the world, with mass vaccination plans approved for the vaccine in the population.

The Medicines and Healthcare Products Agency in the United Kingdom has approved the COVID-19 BNT162B2 developed by Pfizer (USA) and Bio N-Tech (Germany), the COVID-19 vaccine developed by AstraZeneca, and the COVID-19 mRNA 1,273 developed by Moderna for emergency use (8–10). The FDA of the United States has approved Pfizer COVID-19 vaccine, Moderna COVID-19 vaccine and Janssen COVID-19 vaccine for emergency use (11, 12). In addition to Estonia, Malta and Norway, at least 26 countries were using a combination of AstraZeneca, Moderna and Pfizer COVID-19 vaccine (13). And countries including Pakistan, Morocco, Hungary, Bolivia, Nepal and Argentina had approved the emergency use of the COVID-19 vaccine developed by China Biotech Corporation. Indonesia, Brazil, Turkey and Chile are among the countries that have approved emergency use of the COVID-19 vaccine from Beijing Kexing Biotech Co. Ltd (14). At present, the top 5 countries for total doses of COVID-19 vaccine were the United States (6.129 million), China (4.052 million), the European Union (2.66 million), the United Kingdom (1.82 million) and India (1.084 million) (15).

## Immunogenicity and safety of existing COVID-19 vaccines

These vaccines have different action mechanisms against COVID-19, leading to different injection procedures, timing and dosages. Most existing COVID-19 vaccines have good immunogenicity and safety, and are widely tolerated by the population. Injection can improve the seroconversion rates (SR) and geometric mean titer (GMT) *in vivo*, which can significantly enhance human immunity against COVID-19 (16, 17). The most common adverse reaction at the injection site were pain, and the most systemic adverse reactions were fatigue and fever. The vast majority of adverse reactions in the vaccinated subjects were mild to moderate and resolved within 48 h of vaccination (18–20). The immunogenicity of the vaccines may increase with increasing dose and its adverse reactions may increase accordingly. At the same time, the injection procedure and age will also affect the effectiveness and safety of the vaccine. The most widely used vaccine is inactivated vaccine, which is the first vaccine in the world to have the results of animal tests made public. After injection of inactivated vaccine, SR is higher than 90%, and the incidence of adverse reactions is <30%. In addition, SR and GMT will increase with the increase of vaccine dose with good immunogenicity and no grade 3 adverse reactions, indicating reliable safety (21). Adenovirus vector vaccine elicits humoral and cellular immune responses to SARS-CoV-2, and enhanced immunity enhances the titer of neutralizing antibodies, achieving a 70.4% response rate in clinical trials with a 0.15% incidence of serious adverse reactions (22). Double dose of RNA vaccine produced a stronger immune response than single dose, and studies showed that 64.5% of participants had at least one or more symptoms after vaccination, 79.7% of them were able to continue their daily life, 12.33% even required temporary leave of absence, and 3.36% required hospitalization. The incidence of adverse reactions in the elderly was lower than that in the young (23). After injection of recombinant spike protein nanoparticles vaccine, SR exceeded 95% and the incidence of serious adverse reactions was about 1.96%. GMT and adverse reactions increased with the increase of vaccine dose (24). Most adverse reactions after vaccination are common and not life-threatening, suggesting that the body's immune system is building protection (25). The current approved COVID-19 vaccines have been proven to be safe and there is some comfort in getting vaccinated. However, long-term observation and more trials are needed to confirm the safety of COVID-19 vaccines due to the current short follow-up period.

## The main reasons why vaccination did not apparently stop transmission of the SARS-CoV-2

### Phase III clinical trials are few and inconclusive

It usually takes at least 8 or even more than 20 years for a vaccine to go from development to market, including pre-clinical studies and clinical trials. Pre-clinical studies usually take 5–10 years for strain screening, strain attenuating, strain adaptation to cultured cell matrix and stability study during subculture. Clinical trials are divided into three stages, phase I, II and III. Different countries have different strict regulations on human clinical trials of vaccines. Phase I clinical trials preliminatively investigate human safety and generally involve dozens to 100 subjects. The phase II trial will focus on dose exploration, preliminary efficacy evaluation and safety in a larger population, which involve several hundred to thousand cases. Phase III clinical trial is randomized, blind, placebo-controlled designs that comprehensively evaluate the efficacy and safety of vaccines, typically involving thousands to tens of thousands of participants. All clinical trials typically take at least 3 to 8 years, and some even more than 10 years. Phase III clinical trials are the basis for registration and approval of vaccines. However, most types of COVID-19 vaccines have not yet undergone or are still undergoing phase III clinical trials, and the available clinical information is not conclusive enough to represent the true situation after vaccination. The main trial stages and basic information of COVID-19 vaccines showed in Table 1. Although some vaccines have undergone phase III trials and have been approved in several countries, the small number of participants in previous trials cannot accurately reflect the specific clinical protective effect and safety of mass injection. In addition, the sample size and indicators of most clinical trials were not comprehensive enough, and only using SR and GMT to measure efficacy. Although they can reflect the immunogenicity of vaccines to a certain extent, there is insufficient evidence to evaluate their effectiveness. In addition, these indicators are temporary which cannot fully reflect the long-term effects of the vaccines. We believed that the positive rate of SARS-CoV-2 nucleic acid is the most powerful indicator of vaccine efficacy, but no vaccine study has reported this so far. And the debate about the fairness of COVID-19 vaccines has been going on for a long time, with many third world countries still struggling to get a vaccine and global vaccination rates low (26). The phase III clinical trials were carried out in a small number of countries, with incomplete population and ethnicity, small sample size and short observation period, so the research results could only serve as a reference to some extent. And according to the current results and experience, vaccination may not completely prevent SARS-CoV-2 infection, it is unlikely that it will stop the transmission. It can only avoid the occurrence of post-infection disease or reduce the severity of the disease. Therefore, although the existing vaccines have good immunogenicity and safety, we still do not know whether vaccination can significantly reduce the positive rate of SARS-CoV-2 infection, protect against COVID-19, and how long the protection will last.

**Table 1:** Current commonly used COVID-19 vaccines.

**Vaccine name**	**Vaccine type**	**Research and development company**	**Current clinical stage**	**Participants number**	**Approved time**	**Approved countries**
CoronaVac	Inactivated vaccine	SinoVac-China	Phase III (being conducted)	600 (Phase II)	February 5, 2021	China, Brazil, Ukraine
BBIBP-CorV	Inactivated vaccine	SinoPharm-China	Phase II	31,000	April 29, 2021	China, United Arab Emirates, Bahrain, Egypt, Australian
BNT16b2	mRNA vaccine	Pfizer-the USA +BioNTech-Germany	Phase II	43,448	December 2, 2020	the UK, Canada, the USA, Bahrain, Saudi Arabia, Mexico, European Union, Japan
mRNA-1273	mRNA vaccine	Moderna-the USA	Phase II	30,000	November 19, 2021	the UK, European Union, Israel, Canada, the USA, Japan
NVX-CoV2373	Recombinant protein vaccine	Novavax-the USA	Phase III (being conducted)	131	December 20, 2021	Put on an emergency use list
ChAdOx1 nCoV-19 (AZD1222)	Adenovirus vector vaccine	AstraZeneca-the UK	Phase II	11,636	February 22, 2021	India, Argentina, the Dominican Republic, Salvador, Mexico, Morocco, the UK, Japan
Ad26.CoV2.S	Adenovirus vector vaccine	Janssen-the USA	Phase III (being conducted)	796 (Phase I/II)	March 1, 2021	Put on an emergency use list
Ad5-nCoV	Adenovirus vector vaccine	CanSinoBIO-China	Phase III (being conducted)	508 (Phase II)	September 16, 2021	China, Ecuador, Argentina, Brazil, Malaysia
Sputnik V	Adenovirus vector vaccine	Gamaleya Center-Russia	Phase III	18,794	February 3, 2021	Russia, Algeria, Argentina, Bolivia, Serbia, Republic of Belarus

### The pertinence of COVID-19 vaccines development lags behind the rate of virus mutation

**Figure 1:**
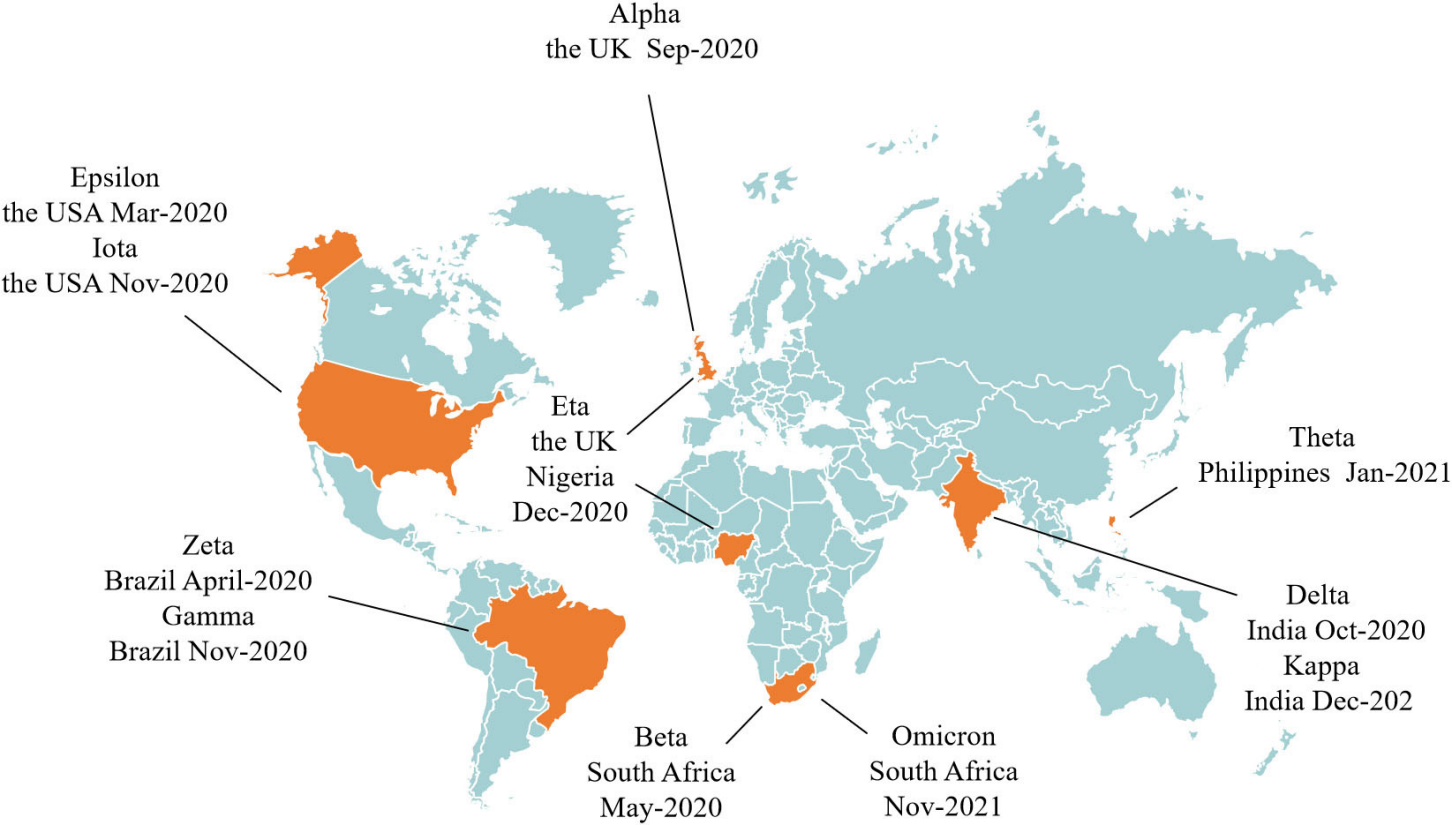
The time and place of discovery of SARS-CoV-2 variants.

SARS-CoV-2 is a single-stranded plus stranded RNA virus with a genome length of about 30kb, which is the virus with the longest nucleic acid chain among the known RNA viruses, it is easy to mutate with the spread of the virus and its mutation may have the potential to affect the pathogenicity of the virus. Whether the COVID-19 vaccines are still effective in SARS-CoV-2 mutation is an important question for vaccine injection. At present, multiple SARS-CoV-2 variants have emerged, Alpha, Beta, Delta, Gamma and Omicron have been reported as variants of concern by WHO, and Epsilon, Eta, Iota, Kappa, Theta and Zeta as variants of interest (Figure 1). These mutations greatly increase the infectivity and infection rate of SARS-CoV-2, and the more the virus spreads, the greater the chance of mutation. Studies have shown that in addition to enhancing viral transmisability, Alpha and Beta also cause widespread mutations in spike genes that are insensitive to neutralization by vaccine-induced and infection-induced antibodies, which is very detrimental to the protection offered by monoclonal antibody therapies and vaccines (27), And the neutralization activity of plasma pair to Beta and Gamma variant strains in convalescent COVID-19 patients at the early stage of the epidemic was significantly reduced, which put patients at risk of reinfection with these variants once they recover. The Delta variant is thought to spread faster than other variants, and serum from individuals who received a single dose of Pfizer or Astrazeneca vaccine had little discernible inhibition of the Delta subtype. With two doses of vaccine, 95% of individuals responded neutrally but had a titer 3 to 5 times lower for Delta than for Alpha (28), and a large number of breakthrough infections were reported after mRNA and adenovirus vector vaccines were administered (29). The rapidly spreading Omicron variant is highly likely to compromise some of the vaccine's protection by affecting the ability of antibodies to recognize the virus and block infection, making the vaccine less effective at preventing infection. The variant is highly contagious, spreading several times faster than the Delta virus and potentially infecting people who are immune to other variants. Recently, preliminary studies suggested that while the protection provided by existing COVID-19 vaccines will not be completely eliminated by Omicron, continuous booster shots were needed to increase immunity to Omicron (30). So the mutated virus may develop resistance to the existing vaccines, weakening the effectiveness of them. The original vaccine does not have antibodies to the mutated virus, reducing the protection of the vaccines to the human body, and the vaccine needs to be improved over time.

Although some clinical trials showed that the serum antibody level after vaccination remain at a high level, scientific research also found that the body antibody could be reduced in different degrees after vaccination 6 months. So, constantly strengthen injection of vaccination are needed to maintain the body antibody levels, and it also can not completely prevent COVID-19 after vaccination (31). Thus, the current vaccines do not provide permanent protection, and repeated booster shots do not guarantee zero risk of SARS-CoV-2 infection. With the spread of COVID-19, more and more animals besides humans have been found to be susceptible to the disease, and the host of the disease has been expanding, increasing the types and number of susceptible animals. A variety of mammals have been infected as COVID-19 hosts through contact with COVID-19 patients and can be passed back to humans, leading to further transmission. In addition, the “wild” host expansion of COVID-19 is also under way, and frequent human wildlife research will increase the risk of people contracting COVID-19. We do not yet know how many host animals are susceptible to COVID-19, which makes screening difficult. The continuous expansion of COVID-19 hosts accelerates the mutation and spread of COVID-19 in silence, which poses an unknown threat to humans (32). Currently, vaccine development is struggling to keep up with the rate of virus mutating, and new strains may appear just as an effective vaccine against the latest variant is developed. The constant mutation of the virus is not only a key factor in the current COVID-19 pandemic, but also poses a huge challenge to vaccine development. SARS-CoV-2 variants are still evolving constantly that could ultimately render our current therapeutic and preventive interventions for COVID-19 ineffective. These require us to stop transmission of the virus as quickly as possible, rapidly develop new vaccines and accelerate vaccine application. It is better to develop a universal effective vaccine against all novel coronavirus strains to prevent the virus from mutating and spreading.

### The long-term adverse reactions after injection of COVID-19 vaccines are unknown and the injection hesitation phenomenon exists

Some serious adverse reactions and side effects associated with the vaccine may occur long after the vaccine has been put into use. A common toxic adverse reaction of vaccines is the anti-dependent enhancement (ADE), which is the significant increase in the ability of certain viruses to replicate or infect with the help of existing non-neutralizing or poorly neutralizing antibodies. However, the time between the discovery of SARS-CoV-2 and the development of a COVID-19 vaccine has not been long enough, and the vaccine has not been available for use for <1 year. The role of ADE in SARS-CoV-2 is unclear, but it has been reported in other coronavirus vaccines (33, 34). There had also been some clinical reports of serious adverse events after vaccination, although the researchers believed that these were not obviously related to the COVID-19 vaccine itself, it were due to comorbidities of the participants (35). The potential adverse reactions caused by the vaccines are still unclear, and the long-term adverse reactions are even more unpredictable. So many people are reluctant or delayed to get vaccinated against COVID-19, and there is a phenomenon of vaccine hesitation in society (36), which reduce vaccine coverage rate, make it difficult to achieve herd immunity, so the COVID-19 vaccine cannot play the desired role.

### The antibodies of the elderly decrease rapidly after vaccination and the vaccines have little protective effect on them

Aging is an independent risk factor for COVID-19 death and severe case, the majority of COVID-19 deaths and severe cases are in the elderly, and patients over 60 years of age with severe comorbidities are shown to have a higher risk of death (37), As populations age, older people are growing rapidly in all countries, which makes it even more important for them to be vaccinated against COVID-19. However, due to various reasons such as immune aging, the antibodies in the elderly decreases rapidly or even disappears after vaccination, which limits the protective effect of the vaccine on them, so they are still at high risk of COVID-19 and severe illness (38). Studies have shown that the effectiveness, immunogenicity and antibody duration of vaccination in the elderly are lower than those in the young (39, 40). And vaccination priority for adults 20 to 49 years of age minimizes cumulative morbidity, but when vaccination priority is given to adults over 60 years of age, mortality and life loss are minimized in most cases (41). Because most vaccines evaluated in early clinical trials have been administered in healthy young people, immunization programs that work well in healthy people may not be appropriate for older people. In addition, the elderly mostly have comorbidities, so most vaccines are not classified as suitable for vaccination due to these uncertain factors, which brings certain difficulties to the protective effect of vaccination on the elderly.

### Injections in children may cause some diseases to worsen but children are more likely to spread the infection

In terms of the development of the global epidemic, the proportion of cases in children has increased significantly, the age of infected children is also decreasing. Although the incidence and severity of COVID-19 remains lower than in adults, the child population may play an important role in the spread of the virus. Studies have shown that the SARS-CoV-2 load in the nasopharynx of children patients is equal to or even greater than that of adult patients, and the children have more aggregation activities, which makes the infection more easily spread (42). This showed that children should be a priority target for vaccination and need greater protection. If not vaccinated, almost everyone, including young children, is at risk of contracting SARS-CoV-2 at some point in their lives. While most children with COVID-19 experience asymptomatic or mild symptoms, some become severe and a small percentage die. But it found that children after injection of COVID-19 vaccines may aggravate their original diseases, or produce adverse reactions, such as tic disorder, attention deficit hyperactivity disorder and so on, so some parents are hesitant to vaccinate their children (43). In addition, the recommended age of vaccination for most vaccines is still 18 years and older. We believe that the reason why children are not recommended to be vaccinated is related to the uncertain safety. Vaccines go through multiple stages of research including safety evaluation and phase I/II/III clinical trials before they can be registered on the market. It usually takes years to decades from development to marketing. Because there is no clear safe and reliable vaccine, ensuring the safety of children is the top priority. The vaccine research is still in the experimental stage, and even the common vaccine is tested in adults first. The immune response caused by the COVID-19 vaccine is complex, and due to the different immune responses of children and adults, there must be many differences from a few months of infants to teenagers. These differences lead to the different doses and times of vaccination of some vaccines, which brings some challenges to the research, development and accurate application of COVID-19 vaccines. But at the current rate of development, the prospects for COVID-19 vaccine development look very promising. We look forward to the early development of a vaccine suitable for children to protect their health and normal life.

## Conclusions and prospects

With the continuous expansion of vaccination coverage, COVID-19 prevention and control has gradually shifted to a strategy based on vaccination and supplemented by drug intervention. At present, there is a cautious and positive trend in global vaccine development for COVID-19. Vaccination plays a part in preventing and controlling COVID-19, which puts us in a good position to combat SARS-CoV-2 infections. While the majority of COVID-19 vaccines have shown good immunogenicity and safety, many uncertainties remain for the future. Due to the short time on the market of COVID-19 vaccines and the lack of observational data, adverse reactions after a long time are still unknown. At present, there are many kinds of vaccines in clinic, with different injection doses and procedures, uncertain tolerance of people of different ages, and vaccine hesitancy and uneven global injection make it difficult to achieve herd immunity in a period of time. COVID-19 vaccines are time-sensitive and protective for a period of time, booster shots are needed to maintain the efficacy of the vaccine over time, but the risk of infection and possible adverse reactions after repeated booster shots remain unclear.

COVID-19 vaccine research and development should be greatly strengthened in the future. In the process of vaccine development, attention should be paid to long-term possible adverse reactions, the specific situation of children and the elderly should be fully considered, so as to develop more safe and effective COVID-19 vaccines with longer protection period and stronger universality, making it more effective at preventing infection and can induce a broad, strong and long-lasting immune response to reduce the need for continuous booster shots. At the same time, in order to prevent SARS-CoV-2 from mutating, genetic changes of the virus should be continuously monitored, efforts should be made to reduce the rate of virus transmission, and vaccines updating should be accelerated to respond to future mutated strains to ensure that the protection level recommended by WHO continues to be provided against infections and diseases caused by other mutated strains that may arise in the future. A comprehensive vaccine safety monitoring system should be established, and large-sample, multi-center, multi-indicator, long-term follow-up clinical trials should be conducted to explore the exact efficacy and safety of COVID-19 vaccines. Accelerating vaccine production and population immunization process, improving vaccination willingness, overcoming vaccination hesitation, and improving fairness and scientific vaccine distribution are the main strategies for controlling the epidemic in the future. In addition, the advanced development concept of universal COVID-19 vaccine can be put forward and implemented, and a class of effective vaccines for all coronaviruses can be found to prevent the continuous mutation and spread of coronaviruses. As most countries are currently only busy stabilizing their own epidemics, which may be another reason for the spread. In order to prevent the outbreak of COVID-19 in a faster and better way, countries should take a global perspective, ensure a synchronized response, constantly narrow the gap, and take the same measures to jointly fight the pandemic. Whether COVID-19 outbreaks again will depend on the vaccination coverage, protective efficacy, safety and durability of COVID-19 vaccines, as well as the rapid response to virus mutations. At present, people around the world are paying attention and actively responding to the epidemic, we believe that victory over COVID-19 will come soon.

## Author contributions

ShZ and Y-PT: conception, design, and initial draft. ZY, Z-LC, S-JY, and SaZ: critical editing of the manuscript. All authors have read and approved the final manuscript.

## Funding

The authors thank grants from Key Research and Development Program of Shaanxi (2019ZDLSF04-05), the National Natural Science Foundation of China (81974522 and 81974584). This research was also financially supported by Subject Innovation Team of Shaanxi University of Chinese Medicine (2019-YL10).

## Conflict of interest

The authors declare that the research was conducted in the absence of any commercial or financial relationships that could be construed as a potential conflict of interest.

## Publisher's note

All claims expressed in this article are solely those of the authors and do not necessarily represent those of their affiliated organizations, or those of the publisher, the editors and the reviewers. Any product that may be evaluated in this article, or claim that may be made by its manufacturer, is not guaranteed or endorsed by the publisher.

## Abbreviations

ADE: anti-dependent enhancement
GMT: geometric mean titer
SR: seroconversion rates
WHO: World Health Organization.
